# Abortion care in women with underlying medical conditions: The role of multidisciplinary team approach in increasing safety of abortion procedures

**DOI:** 10.1002/puh2.113

**Published:** 2023-07-28

**Authors:** Abraham Fessehaye Sium, Sarah Prager, Ferid A. Abubeker, Lucero‐Prisno Don Eliseo, Wondimu Gudu

**Affiliations:** ^1^ Department of Obstetrics and Gynecology St. Paul's Hospital Millennium Medical College (SPHMMC) Addis Ababa Ethiopia; ^2^ Complex Family Planning Division, Department of Obstetrics and Gynecology UW Medicine Seattle Washington USA; ^3^ Department of Global Health and Development London School of Hygiene and Tropical Medicine London UK; ^4^ Faculty of Management and Development Studies University of the Philippines Open University Los Baños Laguna Philippines

**Keywords:** abortion care, abortion care in cardiac disease, abortion care in medical conditions

## Abstract

**Background:**

There are no clear data driving most clinical recommendations for abortion care in women with underlying medical conditions, such as cardiac disease. Current abortion practice in such women is based on limited retrospective studies, mainly case reports and case series. In our institution (a tertiary center in Ethiopia), we practice a multidisciplinary team approach to abortion care for patients with medical conditions.

**Objective:**

Describe the value of a multidisciplinary team approach in abortion care in patients with underlying medical conditions.

**Methods:**

This is a retrospective descriptive analysis of abortion care in women with underlying medical conditions (cardiac and noncardiac medical conditions) over a 5‐year period (November 2016–October 2021) at St. Paul's Hospital Millennium Medical College, in Ethiopia. Data were extracted by reviewing patients’ medical records using a structured questionnaire. Simple descriptive statistics were applied for analysis using SPSS version 23. Results are presented as frequencies and percentages.

**Results:**

Fifteen induced abortion cases in women with underlying medical conditions were analyzed, out of which 11 were in women who were critically ill. The median gestational age was 20 weeks. Ten subjects, 10/15 (66.7%), had a cardiac condition, whereas the rest 5/15 (33.3%) were noncardiac cases. Ten out of the 11 critically ill patients were managed under multidisciplinary team approach, and there were no complications encountered. Out of these, 7/10 (70%) received medication abortion care between 19 and 25 weeks gestation, including 5 cardiac patients with New York Heart Association‐III and IV conditions.

**Conclusion:**

In this study, more than three quarters of women with medical conditions who had abortion care were critically ill, and almost all of them were managed with a multidisciplinary team approach. None of the patients suffered a deterioration of their medical conditions, demonstrating the utility of incorporating a multidisciplinary team approach during abortion care for such cases.

## Graphical abstract

– There are no clear data supporting most clinical recommendations for abortion care in women with underlying medical conditions.
– In our study, more than three‐quarters of women with medical conditions were critically ill and managed under mutidisciplinary team approach with no encounter of deterioration of their medical conditions.
– Findings of our study underscore the importance of including mutidisciplinary team approach in abortion care for women with medical conditions.

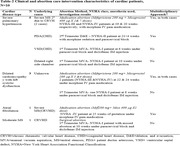

## INTRODUCTION

For all women, delay in seeking abortion care should be avoided, as the safety of abortion somewhat depends on gestational age, even in the first trimester [1]. It should be noted that women with more serious medical problems, such as cardiac disease, also seek abortion services [2, 3]. Avoiding delays for these women is particularly important, as their medical condition may deteriorate with advancing pregnancy. For example, pregnancy‐related physiological changes, such as increased maternal blood volume and cardiac output peak by the end of the second trimester, pose a significant danger to the wellbeing of cardiac patients; hence, extra care is required during abortion care for such patients [2, 4]. Most medication abortion regimens include a prostaglandin, which is vasoactive and leads to vasodilation or vasoconstriction, depending on the target organ's receptors. Limited data exist regarding the cardiovascular effects of misoprostol [5].

According to the Society of Family Planning clinical guidelines, early abortion care and effective postabortion care for women with medical problems reduce pregnancy‐associated morbidity and mortality. However, there is no level A evidence to‐date that supports specific clinical recommendations for a different approach to abortion care in these women [6]. The current available evidence is based on retrospective review studies and case reports, which are also too few in number. Bagga et al. reported no major morbidity or mortality in a 12‐year analysis of first‐ and second‐trimester‐induced abortions in women with cardiac disorders [7]. Similarly, an earlier review of 13 critically ill women undergoing abortion at 20–24 weeks reported no complications [8]. In 2006, Hackney et al. reported successful first‐trimester medication abortion in a woman with underlying coronary artery disease and previous myocardial infarction [9]. Another case report described a previously healthy woman who presented with an acute ischemic infarct after receiving a one‐time dose of 1800 μg intravaginal misoprostol for elective abortion at 12 weeks of gestation [10].

Requests for abortion care in the second trimester are common in Sub‐Saharan Africa, with a prevalence as high as 53%–64% [11, 12] reported in recent studies. This is greater than the 10%–15% prevalence reported globally [13]. Moreover, the unmet need for contraception in women with chronic medical conditions (e.g., cardiac disease) in the region is high, which exposes such women to a high risk of unintended pregnancy, which may result in a need for abortion. A recent prospective study from Ethiopia shows that the overall unmet need for contraception among women with cardiovascular disease having follow‐up at a tertiary hospital is 36.0% (95% CI: 30.4–41.5). The study was conducted at St. Paul's Hospital Millennium Medical College (SPHMMC) (one of the leading tertiary hospitals in Ethiopia). A total of 284 subjects were included, and 65% had underlying cardiac problems (congenital heart disease, rheumatic valve disease, coronary heart disease, and cardiomyopathy) [14]. In short, this region of Africa has a high unmet need for contraception among patients with medical conditions, which translates in to a higher risk of unintended pregnancy that could result in abortion, with a higher probability of second‐trimester abortion. Hence, it is imperative that abortion care for patients with medical conditions in this region of Africa is practiced with maximum safety consideration.

Over the last 5 years, SPHMMC (Ethiopia) has introduce various subspecialty level care and postdoctoral fellowship programs. High‐risk pregnancies are often managed jointly with a team of consultants from different subspecialty units. Utilization of a multidisciplinary team management approach during abortion care in patients with underlying medical conditions is one of these joint efforts that has been implemented to improve patient outcomes. This paper describes the role of this multidisciplinary approach in abortion care in women with underlying medical conditions at this tertiary hospital in Ethiopia.

## METHODS

This study is a retrospective descriptive study of safe abortion cases with underlying cardiac and noncardiac medical conditions that were managed at SPHMMC (Ethiopia) from November 2016 to October 2021. This study setting is a tertiary hospital and one of the leading medical colleges in Ethiopia. More than 16 subspecialty and fellowship training programs, including family planning, cardiology, hematology, and nephrology, are offered at the college.

Data on reproductive characteristics, clinical and investigation findings, abortion care methods, and procedure‐related outcomes were extracted using a data‐extraction tool prepared in English. Resident physicians’ evaluation notes, specialists’ evaluation notes, operation notes, and medication chart were reviewed. Through chart review, we retrospectively identified the proportion of critically ill patients who were managed under a multidisciplinary team management approach, any deterioration of medical conditions, occurrence of cardiovascular events, and major abortion related complications as the outcomes. We defined the multidisciplinary management approach as a team of specialists from family planning, maternal fetal medicine, cardiology, nephrology, anesthesiology, and hematology units, depending on the situation, that jointly provide expertise to most safely provide abortion care to critically ill patients with medical conditions.

Critically ill patients are defined in this study as women who were either at risk of developing hemodynamic instability and/or had either organ failure or were at risk of developing organ failure or high risk of bleeding (cardiac disease in New York Heart Association (NYHA) Class II and above, cerebrovascular accident, bleeding disorder, and chronic kidney disease, etc.). We have used the following NYHA classification of heart failure [15]: Class I: No limitation of physical activity, Class II: Slight limitation of physcial activity, Class III: Marked limitation of physical activity, and Class IV: Unable to carry on any physical activity without discomfort.

A formal letter of ethical clearance was obtained from the Institution Review Board (IRB) at SPHMMC for this study. Informed consent was not applicable due to the retrospective nature of our study and not obtained from study subjects. Data were entered into SPSS version 23, and simple descriptive statistics were used for analysis. Results are presented as frequencies and percentages.

## RESULTS

Out of 15 patients with underlying medical conditions who had abortion care, 10/15 (66.7%) had cardiac problems (Table 1), whereas the rest, 5/10 (33.3%), had noncardiac conditions (2 chronic kidney disease cases, 2 bleeding disorder cases, and 1 stroke case). Eleven patients were critically ill, and 10 out of them were managed under multidisciplinary team management approach. The median gestational age was 20 weeks, and the majority, 10/15 (66.7%), were parous. Eight (53.3%) were managed with surgical abortion, whereas the remaining seven (46.7%) were managed with medication abortion. All the NYHA II and above cardiac patients (6/10, 60%) were managed with a multidisciplinary team comprised of a family planning specialist, a cardiologist, and an anesthesiologist (Table 2). None of these patients had a procedure‐related complication, such as hemorrhage, infection, cervical laceration, or uterine perforation. We also did not see any worsening of cardiac status or occurrence of cardiovascular events. A diuretic (Lasix 40 mg IV single dose) was given in the immediate post‐evacuation period, and they were transferred to the medical side for follow‐up after a minimum of 24 h observation in our labor and delivery ward. All cardiac patients had updated echocardiography.

**TABLE 1:** Reproductive and clinical characteristics of abortion care clients with underlying medical conditions, *N* = 15.

Variable	Category	*N*	%
Age	Mean	30.3	
Gestational age	Median	20	
Parity	Nulliparous	5	33.3
	Parous	10	66.7
Underlying medical condition	Cardiac disease	10	66.7
	Chronic kidney disease	2	13.3
	Bleeding tendency	2	13.3
	Stroke	1	6.7
Abortion method	Medication	7	46.7
	Surgical	8	53.3

**TABLE 2:** Clinical and abortion care intervention characteristics of cardiac patients, *N* = 10.

Cardiac disease type	*N*	Underlying cause	Abortion method, NYHA class, anesthesia used, investigation results	Multidisciplinary approach?
Severe pulmonary hypertension	**5**	Severe MS 2^0^ due to CRVH (two cases)	*Medication abortion (mifepristone 200 mg + misoprostol 400 μg single dose for one case and seven doses for the other)*	Yes, in both cases
			NYHA‐III and NYHA‐IV patients at 19 and 20 weeks, respectively, with morphine IV pain medication	
		PDA (CHD)	Second‐trimester D&E—NYHA‐II patient at 21 + 4 weeks with morphine sedation and paracervical block	Yes
		VSD (CHD)	First‐trimester MVA‐NYHA‐I patient at 6 weeks under paracervical block and diclofenac IM injection	No
		Dilated right side chamber	First‐trimester MVA‐NYHA‐I patient at 10 weeks under paracervical block and diclofenac IM injection	No
Dilated cardiomyopathy with left systolic dysfunction	**3**	Unknown	*Medication abortion (mifepristone 200 mg +* *misoprostol 200 μg × 3 and 4 doses)*	Yes, in both cases
			Two patients (NYHA‐III and NYHA‐IV) at 22 and 24 weeks under morphine IV pain medication	
			First‐trimester MVA‐NYHA‐I patient at 8 weeks under paracervical block and diclofenac IM injection	No
Atrial fibrillation	1	Severe MS (CRVHD)	*Medication abortion (Mif 200 mg + Miso 400 μg × 1 dose*)	
			NYHA‐IV patient at 25 weeks of gestation under morphine IV pain medication	Yes
Moderate MS	1	CRVHD	*Surgical abortion*	
			First‐trimester MVA‐NYHA‐I patient with missed abortion at 10 weeks under paracervical block and diclofenac IM injection	No

Abbreviations: CRVH, chronic rheumatic valvular heart disease; CHD, congenital heart disease; D&E, dilation and evacuation; MVA, manual vacuum aspiration; MS, mitral stenosis; PDA, patent ductus arteriosus; VSD, ventricular septal defect; NYHA, New York Heart Association Functional Classification.

Five cardiac patients (Table 2), 5/10 (50%), had severe pulmonary hypertension with different underlying causes (severe mitral stenosis secondary to chronic rheumatic valvular heart disease, patent ductus arteriosus secondary to congenital heart disease, ventricular septal defect secondary to congenital heart disease, and dilated right side chamber). Three of these five patients presented in the second trimester (at 19–21 weeks of gestation). Two cases were managed with medication abortion, whereas the third one received surgical abortion care. After severe pulmonary hypertension, dilated cardiomyopathy with left systolic dysfunction was the most frequently observed cardiac disease. Three patients presented with such disease at 10, 22, and 24 weeks of gestation, in NYHA‐I, III, and IV conditions, respectively, and all of them were managed medically. Among the other few cases was one patient who had atrial fibrillation (rate‐controlled on digoxin 0.125 mg orally daily and on anticoagulation medication‐warfarin 2.5 mg). She had underlying rheumatic valvular heart disease (severe mitral stenosis) and presented in NYHA class‐IV. She was managed with second‐trimester medication abortion at 25 weeks gestation (mifepristone 200 mg + misoprostol 400 μg sublingual single dose) as the gestational age was too advanced for second‐trimester surgical abortion through dilation and evacuation (D&E).

Among the noncardiac patients analyzed (Table 3), four (80%) patients (two chronic kidney disease patients and two patients with bleeding disorders) were managed with a multidisciplinary approach. In both chronic kidney disease patients, a combination of overnight intracervical Foley catheter placement, mifepristone 200 mg oral and misoprostol 400 μg sublingual single doses were provided at gestational ages of 20 and 24 weeks for the indications of acute graft rejection and missed abortion, respectively. There were no complications. Analysis of the two patients with bleeding disorders shows that both were managed with second‐trimester D&E, and in both cases, the indication for safe abortion was missed abortion. Overnight laminaria with adjuvant mifepristone (200 mg oral) and misoprostol (400 μg sublingual) was used to prepare the cervix. In the second patient, warfarin was discontinued 48 h prior to the procedure and replaced with heparin 5000 IU subcutaneous injection twice per day (the morning dose of heparin on the procedure day was omitted). One patient had a stroke, and second‐trimester D&E was planned after cervical preparation with laminaria, mifepristone 200 mg oral, and misoprostol 400 μg sublingual but the patient expelled before the D&E procedure. There were no procedure related complications or cardiovascular events. Multidisciplinary approach was not incorporated in the management of this case as this patient presented to our hospital before the family planning fellowship came into existence, and before the practice of using a multidisciplinary approach for abortion care became routine.

**TABLE 3:** Clinical and abortion care intervention characteristics of noncardiac patients, *N* = 5.

Medical condition	*N*	Underlying causes	Abortion method, specific diagnosis, anesthesia used, investigation results	Multidisciplinary approach?
CKD	**2**	DM and Chronic HTN (in both cases)	*Medication abortion (Overnight Foley + mifepristone 200 mg + misoprostol 400 μg × 1 dose)*	
			Patients at 20 and 24 weeks, no use of pain medications	Yes, in both cases
			1. Patient with end‐stage renal disease on dialysis (serum creatinine = 8.9 and K^+^ = 5.34) 2. Kidney transplant recipient with acute transplant rejection (24‐h urine protein = 3985 with normal serum creatinine)	
Hematologic (bleeding disorder)	**2**	1.Liver cirrhosis	Second‐trimester D&E under spinal anesthesia in both patients, at 18 and 24 weeks, respectively	Yes, in both cases
		2.Antiphospholipid syndrome	1. Decompensated chronic liver disease(CLD), large volume of ascites, low platelet count, missed abortion Platelet count = 80,000, coagulation profile; PT = 17.3, PTT = 34.3, INR = 1.35 albumin = 1.93, GOT = 35.8, GOT = 23.5, serum bilirubin = 1.93 2. Acute DVT on warfarin treatment (7.5 mg daily) + missed abortion coagulation profile; PT = 12.7, PTT = 58.6, INR = 0.93	
Cerebrovascular accident	1	Vasculitis	Second‐trimester D&E was planned but expulsion occurred after two laminaria were inserted for cervical preparation (*mifepristone 200 mg orally and misoprostol 400 μg × 1 sublingual were also given)*	No
			Brain MRI finding: Left middle cerebral artery territory subacute infarction likely post‐vasculitis	

Abbreviations: CKD, chronic kidney disease; DVT, deep vein thrombosis; D&E, dilation and evacuation; PT, prothrombin time; PTT, thrombin time.

## DISCUSSION

In this study, 10 out of the 11 critically ill patients were managed successfully with a multidisciplinary management approach, without any abortion procedure‐related complications or clinical deterioration of medical conditions. Seventy percent (7/10) of those managed with the multidisciplinary approach received medication abortion (combined regimen with misoprostol 200 and 400 μg sublingual every 3 h, 4–7 doses in some cases) at gestational ages of 19–25 weeks, including five cardiac patients with NYHA‐III and IV status. Cardiac disease complicates approximately 1%–3% of pregnancies and is responsible for 10%–15% of maternal mortality [16, 17]. Complications noted to have occurred in previous large studies include pulmonary edema from congestive heart failure (12.3%), cardiac arrhythmias (6%), thromboembolism (1.9%), angina (1.4%), hypoxemia (0.7%), infective endocarditis (0.5%), and overall maternal mortality rate of 2.7% [18]. NYHA Class III & IV, obstruction of the left heart, prior cardiac events, such as arrhythmia, and ejection fraction <40% are the known predictors of a cardiac event [19, 20, 21]. All cardiac patients analyzed in this study had one or more predictors of a cardiac event. For example, 50% (5/10) of the cardiac patients were in NYHA Class III or IV status, and all of them received the standard misoprostol dose as per the protocol for second‐trimester medication abortion at our institution, and no cardiac event or worsening of heart failure occurred.

WHO 2012 abortion care guidelines recommend the use of clinical judgment in administering misoprostol to cardiovascular patients during medication abortion and put it as a relative contraindication in such patients [22]. However, limited data are available on the use of prostaglandins in women with cardiac disease. There is also very little data about induced abortions in women with heart problems, and coordinated care by the cardiologist, anesthetist, and gynecologist in an institutional setting is likely to lessen the risks in such women. Bagga. et al observed that only 14% (9/65) of his study subjects received medication abortion. Out of these nine patients that received misoprostol, only two were high‐risk patients—NYHA >II [7]. Earlier, a one‐year review of 13 cases of second‐trimester termination at 20–24 weeks by dilation and evacuation, with a wide range of diagnoses, including cardiac disease reported no complications [8].

Although D&E is preferable in cases with underlying medical conditions, as the timing of the procedure can be planned for maximal use of facilities and staff (the patient is spared the possibility of an unattended delivery), the length of the abortion process is minimal, and incidence of infection and retained placenta is lower), the abortion care should be as person‐centered as possible, and the patient's preference of abortion method should be considered. It should be also noted that the safety of D&E declines with increasing gestational age (especially above 20 weeks, and in patients with underlying medical conditions, who are often very sensitive to abortion‐related complications). These were the reasons for managing all the NYHA‐III and IV cardiac cases in this study with medication abortion. There is no evidence that mifepristone or misoprostol affects hemostasis. For women with a bleeding concern, surgical management offers direct observation and immediate uterine evacuation and less often leads to a delayed hemorrhage [23]. Among the noncardiac cases analyzed in our study, two women with bleeding disorders were managed with second‐trimester dilation and evacuation under spinal anesthesia. A multidisciplinary management approach (composed of a hematologist, family planning, and anesthesiologist) was applied in both cases with no encounter of procedure and non‐procedure related complications.

Mifepristone undergoes hepatic and renal metabolism and is not advisable in patients with severe hepatic impairment or renal failure [24]. From the two patients with bleeding disorder in the present study, one patient had decompensated chronic liver disease. Mifepristone and misoprostol were provided as adjuvant to laminaria for cervical preparation before D&E for both patients. The other patients that were managed under the multidisciplinary team approach were two patients with chronic kidney disease who also received misoprostol and mifepristone, but as a regimen of second‐trimester medication abortion along with overnight intracervical Foley catheter placement. Similarly, there were no complications encountered in those cases. The use of mifepristone in patients with liver and kidney disease in our study was weighed against the risk of significant bleeding from cervical laceration and finally decided to benefit the patients from the cervical softening effect of mifepristone. When patients requiring anticoagulation medication(s) seek abortion, providers are faced with the clinical decision of whether to interrupt or modify therapy. Reversal of anticoagulation may decrease bleeding but increase the risk of thromboembolism, particularly during pregnancy. Women with a high risk of thrombosis maintained on warfarin may be transitioned to heparin, which can be held for surgery, and then warfarin may be restarted [25]. Although this approach is time‐consuming and complex, it was the management strategy utilized in our study in the management of the patient with deep venous thrombosis (DVT) who was on warfarin and needed an abortion.

Though our study presents these interesting results, retrospective and non‐comparative study design and small sample size allocation are its main limitations. Lack of long‐term follow‐up on outcomes and analysis of patient acceptability of the methods of abortion in a form of qualitative data are the other limitations of the present study. Our study demonstrates three important future research implications. First, future comparative studies could determine if there is an added value to a multidisciplinary approach to abortion in patients with a medical complications versus the current abortion care protocol for such patients. Second, identifying which method of abortion is safest in patients with underlying medical conditions remains a research gap to be explored further. Third, second‐trimester abortion in such patients, specifically later medication abortion, is not well‐studied and should be explored through prospective studies.

## CONCLUSION

Abortion care in women with underlying medical conditions was completed with no further deterioration of their medical conditions nor any encounter of procedure complications. More than three quarters of these women were critically ill, and almost all of them were managed under multidisciplinary team approach, supporting the helpful role this approach plays in improving patients’ safety throughout the abortion process. We recommend future comparative studies to further clarify the utility of this approach.

## AUTHOR CONTRIBUTIONS

Conceptualization; data curation; formal analysis; investigation; methodology; writing—original draft; writing—review and editing: Abraham Fessehaye Sium. Data curation; formal analysis; methodology; supervision; writing—original draft; writing—review and editing: Sarah Prager. Supervision; validation; writing—original draft; writing—review and editing: Ferid A. Abubeker and Lucero‐Prisno III Don Eliseo. Conceptualization; formal analysis; investigation; methodology; validation; writing—original draft: Wondimu Gudu.

## CONFLICT OF INTEREST STATEMENT

Authors have no financial nor nonfinancial competing interests. Abraham Fessehaye Sium is an Editorial Board Member, and Don Eliseo III Lucero‐Prisno is the Editor‐in‐Chief of Public Health Challenges and also coauthors of this article. They were excluded from editorial decision‐making related to the acceptance of this article for publication in the journal.

## FUNDING INFORMATION

This research did not receive any funding for its conduct or publication.

### ETHICS STATEMENT

Ethical clearance was obtained from St. Paul's Hospital Millennium Medical College IRB. The ethical clearance did not require informed consent from the study subjects.

## Data Availability

All data generated or analyzed during this study are included in this published article.
